# Age related human T cell subset evolution and senescence

**DOI:** 10.1186/s12979-019-0165-8

**Published:** 2019-09-11

**Authors:** Mingde Li, Danlin Yao, Xiangbo Zeng, Dimitri Kasakovski, Yikai Zhang, Shaohua Chen, Xianfeng Zha, Yangqiu Li, Ling Xu

**Affiliations:** 1 Department of Hematology, First Affiliated Hospital; Institute of Hematology, School of Medicine; Key Laboratory for Regenerative Medicine of Ministry of Education, Jinan University, No.601 West of Huangpu Avenue, Guangzhou, 510632 China; 2 Department of Clinical Laboratory, First Affiliated Hospital, Jinan University, Guangzhou, 510632 China; 3 The Clinical Medicine Postdoctoral Research Station, Jinan University, Guangzhou, 510632 China

**Keywords:** Stem cell memory T cell, Central memory T cells, Effector memory T cells, Ageing, Immunosenescence

## Abstract

T cells are fundamental effector cells against viruses and cancers that can be divided into different subsets based on their long-term immune protection and immediate immune response effects. The percentage and absolute number of these subsets change with ageing, which leads to a reduced immune response in older individuals. Stem cell memory T cells (T_SCM_) represent a small population of memory T cells with enhanced proliferation and differentiation properties that are endowed with high potential for maintaining T cell homeostasis. However, whether these cells change with ageing and gender remains unknown. Here, we assayed the distribution of T_SCM_ and other T cell subsets in peripheral blood from 92 healthy subjects (44 females and 48 males) ranging from 3 to 88 years old by flow cytometry. We found that CD4+ and CD8+ T_SCM_ in the circulation have relatively stable frequencies, and the absolute number of CD8+ T_SCM_ decreased with age; however, the ratio of T_SCM_ to the CD4+ or CD8+ naïve population increased with age. Unlike the obvious changes in other T cell subsets with age and gender, the stable level of T_SCM_ in peripheral blood may support their capacity for sustaining long-term immunological memory, while their importance may increase together with ageing.

**Electronic supplementary material:**

The online version of this article (10.1186/s12979-019-0165-8) contains supplementary material, which is available to authorized users.

## Background

Immunosenescence is a complicated process. One discernible alteration is the number and composition of the different types of lymphocytes in the circulation, particularly T cells [1–3]. Conventionally, antigen-exposed T cells have been divided into central memory T (T_CM_) cells (CD45RO + CCR7+), effector memory T (T_EM_) cells (CD45RO + CCR7-), and effector T (T_EF_) cells (CD45RO-CCR7-). With ageing, continuous antigen stimulation and thymic involution lead to a shift in the T cell subset distribution from naïve T cells to T_CM_, T_EM_, and T_EF_ [4]. This process is accompanied by the loss of expression of co-stimulatory molecules, such as CD27 and CD28. The results of these changes are likely to be associated with increased susceptibility to infections, autoimmune disorders, chronic diseases, cardiovascular disease, and even cancers [5–9]. CD28, an important T cell co-stimulatory receptor, is responsible for T cell activation, proliferation, and survival. The accumulation of CD28- T cells, which mainly contribute to age-associated changes in T cells, is associated with a reduced overall immune response to pathogens and vaccines [5, 10].

Recently, a new memory T cell subset, stem cell memory T cells (T_SCM_), has been detected in humans. This subset was identified based on expression of the surface markers CD95 and CD28 on the CD45RO- CCR7+ T cell subset [11]. Compared with other memory T cell subsets, T_SCM_ demonstrates a faster response to antigen stimulation, preferentially survive after the elimination of antigens, stably persist for a long period of time, and reconstitute the entire peripheral T cell population with a small number of cells [12–14]. For instance, Marraco and colleagues found that a yellow fever virus (YFV)-specific T_SCM_ population could stably maintain for more than 25 years in a man who received vaccination [15], while Costa del Amo also identified T_SCM_ in the circulation possessing self-renewal capacity and clonal longevity, which are necessary for sustaining long-term immunological memory [16]. These studies supported that T_SCM_ should play an important role in sustaining peripheral T cell homeostasis; however, whether T_SCM_ could change with ageing has remained unclear. Thus, in this study, we analyzed age-related changes by comparing the CD4 and CD8 compartments in individuals with ageing throughout lifespan.

## Results and discussion

### The overall T cell reservoir decreases with age accompanied by an increase in the CD4 to CD8 ratio in the circulation

**Fig. 1:**
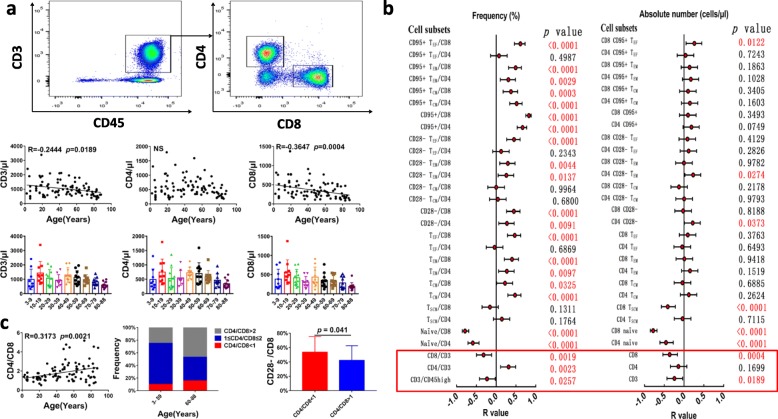
Peripheral T cell reservoirs decrease with age accompanied by an increase in the ratio of CD4 to CD8 cells. **a** The gating strategy for the T cell populations is shown. CD3 T cells were gated from the CD45 high population, CD4 and CD8 T subsets were gated from the CD3+ population. The total CD3 and CD8 but not CD4 T cell numbers decreased with age, but the difference between adjacent age groups is not significant; **b** Correlation and regression analysis of different T cell subsets and ages were calculated. The left represents the frequency, and the right represents the absolute number. The red points and bars represent the R-value and 95% confidence interval of the regression equation, and the *P* value to the right of the figure indicates the statistical significance of each subset. **c** The ratio of CD4 to CD8 increases with age, and three types of CD4/CD8 ratios (> 2; 1–2; < 1) have different frequencies in the young (3–59 years) and old (60–88 years) cohorts compared with an age-matched population of normal CD4/CD8 T cells. The group with the inverted CD4/CD8 ratio has a higher percentage of CD28- cells in the CD8 subsets

Immunosenescence is a multifactorial phenomenon that affects all compartments of the immune system. T cells are dramatically affected by ageing [3]. Based on age, we divided samples into 9 age groups with a span of 10 years. The results demonstrated that the absolute numbers of CD3+ (*P* = 0.0189) and CD8+ (*P* = 0.004) but not CD4+ (*P* = 0.1699) T cells linearly decline with age, and differences between adjacent groups are not significant (Fig. 1a), indicating that the peripheral T cell reservoir gradually decreases with age, which is particularly obvious for the CD8+ T cell subset.

Due to the rapid decrease in CD8+ T cells with age, the ratio of CD4 to CD8 (CD4/CD8) cells increased in the cohorts (Fig. 1b red line box and C). Vasson et al. performed a comparative analysis of 300 healthy individuals from France, Austria, and Spain and found that France and Spain had a decreased CD4 to CD8 ratio with increasing age, and our data were most similar to the results from Austria. These authors thought it was possibly related to diet [17]. However, there was no information from Asia to compare relative differences. The distribution of the CD4/CD8 ratio in our study is shown in Fig. 1c, and there is a higher percentage of persons in the over 60 age group with a CD4/CD8 ratio in excess of two. Whether the increased CD4/CD8 ratio was related to the proliferation of regulatory T cells (Treg) and/or Th2 cells in older people remains unknown. As previously defined, an inverted CD4/CD8 ratio (< 1:1) is an immune risk factor for almost any age, and an inverted CD4:CD8 ratio is related to fewer B cells, expansion of late-differentiated or senescent T cells (CD8 + CD28-), and higher human cytomegalovirus (HCMV) seropositivity [1, 18]. J Strindhall et al. reported that 8.0% of people aged 20–59 years have an inverted CD4/CD8 ratio, while 15.6% of those aged 60–90 years old have an inverted ratio [19]. These numbers are similar to our results where we also found a higher percentage of CD28- T cells in people with an inverted CD4/CD8 ratio in comparable age groups (Fig. 1c). In terms of HCMV infection, it was reported that the latent infection (IgG Seropositivity) rate in the Chinese population is more than 90%, and even reaches 97.03% in Shanghai province (One of the first-tier cities of China) over age 25 [20]. We did not test for the human cytomegalovirus (HCMV) infection in these cohorts, but data from another project from our team showed that 36 out of 37 healthy individuals were seropositive for HCMV IgG (unpublished data). Based on these data, it is hard for us to analyze whether there are differences between HCMV infected and noninfected cohorts.

### An age-related T cell shift in distribution and T_SCM_ homeostasis

**Fig. 2:**
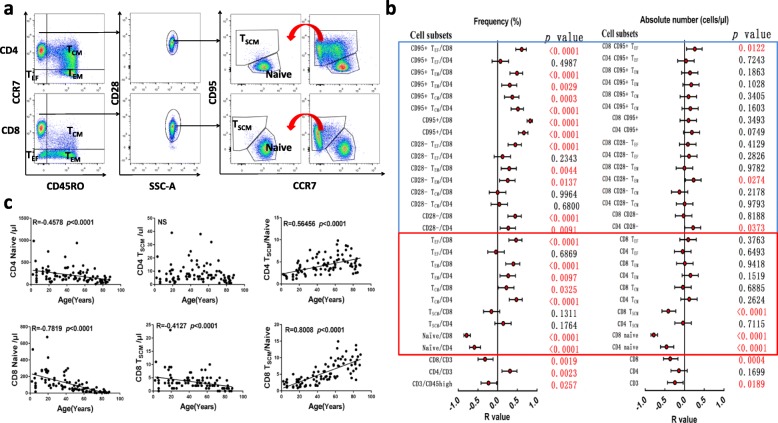
Age-related shift in T cell distribution and T_SCM_ homeostasis. **a** The gating strategy of the T cell subsets populations T_CM_ (CCR7 + CD45RO+), T_EM_ (CCR7-CD45RO+), and T_EF_ (CCR7-CD45RO-), which were gated from CD4 and CD8 T subsets; naïve T cells (CD28 + CD95-) and T_SCM_ cells (CD28 + CD95+) were gated from the CCR7 + CD45RO- T cell subset according to the expression of CD28 and CD95. The curves and red arrow represent the gate to be copied to gate the expression of CD95 and CCR7 on CD4+ or CD8+ T cells. **b** Correlation and regression analysis of different T cell subsets and ages were calculated. The left represents the frequency, and the right represents the absolute number. The red points and bars represent the R-value and 95% confidence R-value of the regression equation. The *P* value to the right of the figure indicates the statistical significance of each subset, and the red box represents the frequency and absolute number of naïve, T_SCM_, T_CM_, T_EM_, and T_EF_, and the blue box represents the relationship between the expression of CD28- and CD95+ and age in the above subsets; **c** While the absolute numbers of CD4 and CD8 naïve and CD8 T_SCM_ but not CD4 T_SCM_ decrease with age, the ratio of CD4 T_SCM_ to CD8 naïve and CD8 T_SCM_ to naïve cells linearly increased with age

The gating strategy for T cells ranging from naïve to T_EF_ cells is shown in Fig. 2a. Consistent with a previous study [4], our data also verified that the proportion of naïve T cells in the CD4+ and CD8+ T cell subsets sharply decrease with ageing, particularly for the CD8 population (Fig. 2b). With the exception of CD4+ T_EF_ cells, the percentage of T_CM_, T_EM_, and T_EF_ cells in the CD4+ and CD8+ T cell subsets accumulated with age (Fig. 2b left, highlighted by red line box). For the absolute numbers in the different T cell subsets, with the exception of a decrease in CD4+ and CD8+ naïve T cells with age, there was no difference in the other subsets (Fig. 2b right, highlighted by red line box). Moreover, the decrease in naïve T cells could be found both in the percentage and absolute number level, indicating that ageing has quite a large impact on the homeostasis of naïve T cells. Due to the erosion of the thymus beginning at approximately age 20, less naïve T cells can be produced, and the increasing antigens that have been encountered and infections that have occurred during the lifespan contribute to the differentiation of naïve T cells into more differentiated T cell subsets. When examining different memory T cell subsets, only the percentage of changes can be detected without apparent changes in absolute cell number, and this is partially due to the diversion in the subsets analyzed. In addition, the decrease in total CD8 T cell number further diluted the change in cell number for each subset. These results indicate that both the percentage and number of naïve T cells are physiologically related to ageing, while the composition of CD4 and CD8 T cell subsets also reflect the immune situation in individuals to different degrees.

T_SCM_ is a newly defined T cell subset with the capacities of self-renewal and differentiation into various memory/effector subsets, and this cell type provides a potential reservoir for T cell memory throughout life [21, 22]. In our previous studies, we found a lower proportion of T_SCM_ in patients with acute or chronic myeloid leukemia compared with age-matched healthy individuals [7, 8]; however, little is known about the T_SCM_ changes with age. Here, we found that T_SCM_ cells account for 1.27 ± 0.55% of the CD4 and 0.98 ± 0.53% of the CD8 population, and the absolute numbers of CD4+ and CD8+ T_SCM_ cells in peripheral blood were 7.97 ± 7.32/μl and 3.44 ± 3.14/μl, respectively. The absolute number of CD8+ T_SCM_ decrease with age (Fig. 2b and c). What is interesting is that although the absolute number of naïve T cells sharply decrease with age, the ratio of CD4+ T_SCM_/CD4+ T_N_ and CD8+ T_SCM_ /CD8+ T_N_ cells linearly increase, particularly for CD8+ T_SCM_ (Fig. 2c). These results indicate that CD4+ T_SCM_ are more stable than CD8+ T_SCM_, which may explain the slower senescence of the CD4+ T cell subset compared with the CD8 population. Furthermore, although the number of CD8+ T_SCM_ decreased with age, the rate of decrease was slower than that of the naïve population. Considering thymus erosion and naïve T cell contraction together with ageing [23], it is reasonable to suspect that T_SCM_ may provide a large contribution to maintaining the homeostasis of peripheral T cell subsets during ageing due to their strong self-renewal and differentiation ability. It appears that the homeostasis of the CD4 population is more stable than that of the CD8 population due to the stable number of CD4+ T_SCM_ in one’s lifetime.

We also analyzed changes in the co-stimulatory molecules CD28 and FAS (CD95) on the T cell subsets with age. As shown in Fig. 2b left (highlighted by a blue line box), the percentages of the CD28- and CD95+ T cell subsets in the CD4 and CD8 populations accumulated with age, but only the CD4 + CD28- T cells had an increase in absolute number. In addition, the increase in different sub-populations demonstrated different characteristics where it appears that the CD4 T_EM_ subset has more profound accumulation of CD28- T cells, while the CD8 T_EF_ subset has a more apparent increase in CD95+ T cells (Fig. 2b left and right, highlighted by a blue line box), which may be due to the change in both percentage and absolute number. Considering that CD28 plays an important role in T cell proliferation and activation, the accumulation of CD28- CD4+ T cells alone with ageing may be an adverse indication for older individuals. In addition, CD95 (Fas) can lead to apoptosis in target cells when it binds to Fas-ligand (Fas-L); thus, the accumulation of the Fas + CD8 + T_EF_ population may partially explain the sharp decrease in CD8+ T cells with ageing compared with CD4+ T cells.

Studies examining gender-dependent changes demonstrated that females produce higher antibody levels and have a higher number of CD4+ T cells than males [24, 25]. Here, we also analyzed the differences in the T cell subset distribution of three age groups (< 20 years; 20–60 years; > 60 years) between females and males. The results show that males in the 20–60 years group have a higher CD8 naïve T cell proportion and absolute count compared with females (*p* < 0.05), while males in the > 60 years group have a higher proportion of CD4 naïve T cells and a lower proportion of CD95 + CD4 T cells compared with females. We then further compared the differences in all of the subjects based on sex and found similar results where a higher CD8 naïve T cell proportion and absolute count were found for males in comparison with females (*p* < 0.05), while males had a lower proportion of CD8+ T_EF_ and CD28-CD8+ T cells compared with females (Additional file 1: Table S1 and Additional file 2: Table S2). These results are interesting and may indicate slower thymus erosion in males and a higher antigen resistant capacity for males compared with females over age 20; however, larger sample analysis is required in the future to confirm this finding.

**Fig. 3:**
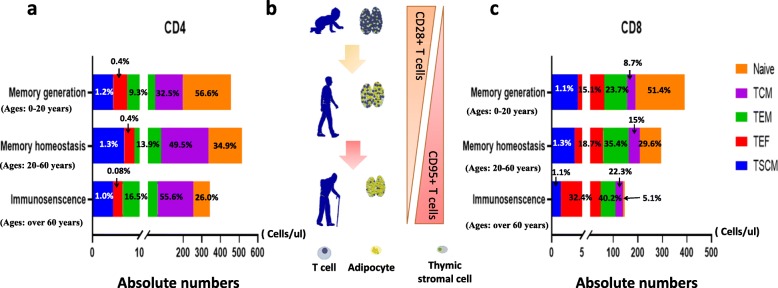
The number and percentage of T cell subsets change with ageing. **a**; **c** Subjects were divided into 3 groups according to three distinct T cell phases: memory generation (ages: 0–20 years, *n* = 19), memory homeostasis (ages: 20–60 years, *n* = 41), and immunosenescence (ages:over 60 years, *n* = 32). **a** The overall lengths of the bars indicate the absolute median count of the CD4 populations in the three phases according to our data. The different parts of each bar represent different T cell subsets, and the median percentage of each population is written in their respective position. **b** Schematic diagram of the ageing contribution to the decrease in T cells and thymic stromal cells and increase in adipocyte in the thymus. This process was accompanied by the accumulation of CD28- and CD95+ T cells in the peripheral blood. **c** The overall lengths of the bars indicate the absolute median count of CD8 populations in three phases according to our data. The different parts of each bar represent different T cell subsets, and the median percentage of each population is shown in their respective positions

Based on changes in memory T cell frequency, pathogen susceptibility and mortality throughout human life, Farber et al. divided an individual’s lifetime into three phases: memory generation (ages 0–20 years), memory homeostasis (ages 20–65 years), and immunosenescence (age > 65 years) [26]. We first summarized the CD4+ and CD8+ T cell changing characteristics in Chinese cohorts based on the three phases (Fig. 3). The total number of CD8+ T cells were more apparently reduced compared with CD4+ T cells across the human lifespan in peripheral blood, and in the sub-population, naïve T cells sharply decreased while T_CM_, T_EM,_ and T_EF_ accumulated with age (Fig. 3a and c). Although the absolute number of CD8+ T_SCM_ decreased with age, the percentage of CD4+ and CD8+ T_SCM_ could maintain a stable level throughout the lifespan. At the same time, CD28- and CD95+ T cells also accumulated, which could result in a loss of activation and proliferation potential in T cells (Fig. 3b). These findings indicate that although T cell reservoirs and function decrease with age, the stable level of CD8+ T_SCM_ frequency may be quite important in older people for maintaining the homeostasis of peripheral T cell memory; however, much work remains to be done in the future to clearly understand the importance of T_SCM_ in humans, such as whether T_SCM_ cells can divide in a self-renewal manner at the clonal level or not?

## Materials and methods

### Sample information

Peripheral blood (PB) samples were obtained from the Department of the clinical laboratory, First Affiliated Hospital of Jinan University. Subjects had a current or recent acute infection, and those with autoimmune disease or diabetes mellitus were excluded. Ninety-two healthy volunteers (44 females and 48 males) ranging from 3 to 88 years old were enrolled. There were 9 different age groups with a span of 10 years. The characteristics of the healthy volunteers are listed in Table 1. The age ranges for each of the groups were as follows: 3–9 years (*n* = 9); 10–19 years (*n* = 10); 20–29 years (*n* = 11); 30–39 years (*n* = 9); 40–49 years (*n* = 10); 50–59 years (*n* = 11); 60–69 years (*n* = 11); 70–79 years (*n* = 10); and 80–88 years (*n* = 11). All procedures were conducted according to the guidelines of the Medical Ethics Committees of the Health Bureau of the Guangdong Province in China, and ethical approval was obtained from the Ethics Committee of the Medical School of Jinan University.

**Table 1:** Characteristics of healthy volunteers

Variable										Total
Age/year	3–9	10–19	20–29	30–39	40–49	50–59	60–69	70–79	80–88	3–88
Number	9	10	11	9	10	11	11	10	11	92
Male	5	6	7	5	5	5	5	4	6	48
Female	4	4	4	4	5	6	6	6	5	44

### Immunophenotyping analysis by flow cytometry

Cell surface staining for flow cytometry was performed using the following antibodies: CD45-APC, CD3-FITC, CD4-APC-H7, CD8-Percp-Cy5.5, CD28-PE, CD95-PE-Cy7, CCR7-BV421, CD45RO-BV510, a BV510 isotype Control, and a BV421 isotype Control. Extracellular staining was performed according to the manufacturer’s instructions. The CCR7-BV421 fluorescent antibody was stained independently. Twenty microliters of absolute count microsphere (Thermo; Cat: C36950) was added to samples for absolute number analysis. Cells were analyzed with a BD Verse flow cytometer (BD, Biosciences, USA), and data analysis was performed using FlowJo software.

### Statistical analyses

All data are represented as median, and statistically significant differences between the different T cell populations and between CD28- and CD95+ T cells were analyzed by the Mann-Whitney U test for nonparametric values. Calculations were performed using GraphPad Prism version 7.00 software and SPSS 23.

## Additional files

Additional file 1: **Table S1.** The difference of T cell frequencies compared between male and female at three age groups. (DOCX 47 kb)

Additional file 2: **Table S2.** The difference T cell absolute number compared between male and female at three age groups. (DOCX 46 kb)

## Data Availability

The datasets used and/or analyzed during the current study are available from the corresponding author upon reasonable request.

## Abbreviations

CMV: Cytomegalovirus
T_CM_: Central memory T cell
T_EF_: Effector T cell
T_EM_: Effector memory T cell
T_N_: Naïve T cell
Treg: Regulatory T cell
T_SCM_: Stem cell memory T cells
