# Supplementary material for: Age related human T cell subset evolution and senescence
Source: Immun Ageing. 2019 Sep 11;16:24. doi: 10.1186/s12979-019-0165-8 (PMC6739976; doi:10.1186/s12979-019-0165-8)
Supplement: Supplementary file 1 — Table S1. The difference of T cell frequencies compared between male and female at three age groups. (DOCX 47 kb) [file 12979_2019_165_MOESM1_ESM.docx]

**Additional file 1: Table S1. The difference of T cell frequencies compared between male and female at three age groups**

| **Age** | **Samples** | | **CD4 (%)** | | **CD8 (%)** | | **Naïve (%)** | | | | **T_SCM_ (%)** | | | | **T_CM_ (%)** | | | | **T_EM_ (%)** | | | | **T_EF_ (%)** | | | | **CD28- (%)** | | | | **CD95+ (%)** | | | |  |
| --- | --- | --- | --- | --- | --- | --- | --- | --- | --- | --- | --- | --- | --- | --- | --- | --- | --- | --- | --- | --- | --- | --- | --- | --- | --- | --- | --- | --- | --- | --- | --- | --- | --- | --- | --- |
| **groups** | **numbers** | |  | |  | | **CD4** | | **CD8** | | **CD4** | | **CD8** | | **CD4** | | **CD8** | | **CD4** | | **CD8** | | **CD4** | | **CD8** | | **CD4** | | **CD8** | | **CD4** | | **CD8** | |  |
| **<20 (years)** |  | |  | |  | |  | |  | |  | |  | |  | |  | |  | |  | |  | |  | |  | |  | |  | |  | |  |
| **males** | **11** | | **51.8** | | **39.9** | | **51.4** | | **49.2** | | **0.83** | | **0.96** | | **33.3** | | **7.53** | | **9.06** | | **22** | | **0.63** | | **15.9** | | **2.32** | | **28.9** | | **41.5** | | **37.2** | |  |
| **females** | **8** | | **48.2** | | **38.5** | | **56.2** | | **43.8** | | **1.07** | | **0.96** | | **28.2** | | **12.9** | | **8.58** | | **19.2** | | **0.89** | | **12.5** | | **1.14** | | **32.7** | | **36.9** | | **35.8** | |  |
| **20-60 (years)** |  | |  | |  | |  | |  | |  | |  | |  | |  | |  | |  | |  | |  | |  | |  | |  | |  | |  |
| **males** | **22** | | **54.1** | | **36.2** | | **33.7** | | ***37.3*** | | **1.19** | | **1.07** | | **44** | | **12.7** | | **13.7** | | **29.8** | | **0.31** | | **14.1** | | **1.78** | | **26.7** | | **54.4** | | **50.9** | |  |
| **females** | **19** | | **58.2** | | **31.7** | | **32.7** | | ***24.9**** | | **1.19** | | **0.83** | | **46.5** | | **18.6** | | **11.2** | | **33** | | **0.53** | | **18.6** | | **3.69** | | **33.2** | | **55** | | **57.4** | |  |
| **>60 (years)** |  | |  | |  | |  | |  | |  | |  | |  | |  | |  | |  | |  | |  | |  | |  | |  | |  | |  |
| **males** | **15** | | **60.7** | | **27.2** | | ***31.2*** | | **8.25** | | **1.36** | | **0.67** | | **52.7** | | **18.5** | | **15.7** | | **38** | | **0.59** | | **28** | | **4.37** | | **50.7** | | ***63.5*** | | **77.1** | |  |
| **females** | **17** | | **55.5** | | **36.1** | | ***18.6**** | | **5** | | **1.09** | | **0.66** | | **55.6** | | **13.6** | | **16.3** | | **34** | | **0.46** | | **36** | | **7.63** | | **49.5** | | ***69.3**** | | **80.7** | |  |
| **Total** |  | |  | |  | |  | |  | |  | |  | |  | |  | |  | |  | |  | |  | |  | |  | |  | |  | |  |
| **Males** | | **48** | | **53.7** | | **35.6** | | **34.5** | | ***33*** | | **1.26** | | **0.95** | | **42.6** | | **11.5** | | **14.2** | | **31** | | **0.52** | | ***16.3*** | | **2.59** | | ***32.9*** | | **55** | | **56.8** | |
| **females** | | **44** | | **53.7** | | **36.5** | | **30.9** | | ***19.9**** | | **1.17** | | **0.77** | | **48.9** | | **14.5** | | **12.7** | | **30.4** | | **0.57** | | **22.2*** | | **4.69** | | ***40.9**** | | **60.4** | | **60.5** | |

****:* Compared with males, *p*<0.05**

**The data are represented as medians, and statistically significant differences between the males and females were analyzed by the Mann-Whitney U test for Nonparametric Tests.**
