# Supplementary material for: Age related human T cell subset evolution and senescence
Source: Immun Ageing. 2019 Sep 11;16:24. doi: 10.1186/s12979-019-0165-8 (PMC6739976; doi:10.1186/s12979-019-0165-8)
Supplement: Supplementary file 2 — Table S2. The difference T cell absolute number compared between male and female at three age groups. (DOCX 46 kb) [file 12979_2019_165_MOESM2_ESM.docx]

**Additional file 2: Table S2. The difference T cell absolute number compared between male and female at three age groups**

| **Age** | **Samples** | **CD4 (/μl)** | **CD8 (/μl)** | **Naïve (/μl)** | | **T_SCM_(/μl)** | | **T_CM_ (/μl)** | | **T_EM_ (/μl)** | | **T_EF_(/μl)** | | **CD28- (/μl)** | | **CD95+ (/μl)** | |
| --- | --- | --- | --- | --- | --- | --- | --- | --- | --- | --- | --- | --- | --- | --- | --- | --- | --- |
| **groups** | **numbers** |  |  | **CD4** | **CD8** | **CD4** | **CD8** | **CD4** | **CD8** | **CD4** | **CD8** | **CD4** | **CD8** | **CD4** | **CD8** | **CD4** | **CD8** |
| **<20 (years)** |  |  |  |  |  |  |  |  |  |  |  |  |  |  |  |  |  |
| **males** | **11** | **467** | **381** | **273** | **234** | **4** | **3** | **114** | **44** | **34** | **75** | **4** | **55** | **11** | **103** | **172** | **141** |
| **females** | **8** | **512** | **468** | **278** | **177** | **6** | **5** | **173** | **40** | **42** | **72** | **5** | **65** | **7** | **112** | **183** | **155** |
| **20-60 (years)** |  |  |  |  |  |  |  |  |  |  |  |  |  |  |  |  |  |
| **males** | **22** | **565** | **336** | **181** | ***116*** | **7** | **4** | **313** | **38** | **81** | **82** | **2** | **34** | **13** | **94** | **329** | **174** |
| **females** | **19** | **585** | **315** | **188** | ***72**** | **6** | **3** | **247** | **46** | **66** | **75** | **3** | **46** | **12** | **98** | **298** | **165** |
| **>60 (years)** |  |  |  |  |  |  |  |  |  |  |  |  |  |  |  |  |  |
| **males** | **15** | **452** | **167** | **137** | **13** | **6** | **1** | **177** | **28** | **73** | **79** | **2** | **43** | **15** | **85** | **261** | **131** |
| **females** | **17** | **416** | **274** | **77** | **16** | **5** | **1** | **243** | **37** | **62** | **62** | **2** | **112** | **26** | **127** | **334** | **224** |
| **Total** |  |  |  |  |  |  |  |  |  |  |  |  |  |  |  |  |  |
| **Males** | **48** | **491** | **297** | **179** | ***91*** | **6** | **3** | **216** | **34** | **66** | **81** | **2** | **42** | **13** | **89** | **285** | **142** |
| **females** | **44** | **491** | **307** | **136** | ***52**** | **6** | **3** | **232** | **45** | **59** | **82** | **3** | **73** | **18** | **119** | **300** | **180** |

****:* Compared with males, *p*<0.05**

**The data are represented as medians, and statistically significant differences between the males and females were analyzed by the Mann-Whitney U test for Nonparametric Tests.**
